# Effects of Failure Acceptance, Entrepreneurial Orientation, and Social Safety Net on Entrepreneurial Intention: A Moderated Mediation Analysis of Korean Employees

**DOI:** 10.3390/bs15010028

**Published:** 2024-12-30

**Authors:** Yu Jin Chang, Arpine Martirosyan, Hae Wen Lim, Jae Wook Yoo

**Affiliations:** 1 Department of Global Business Administration, Anyang University, Anyang 14028, Republic of Korea; parksk@anyang.ac.kr; 2 College of Business Administration, Konkuk University, Seoul 05029, Republic of Korea; arpinemart9@konkuk.ac.kr; 3 Department of Smartcity Engineering, Anyang University, Incheon 23038, Republic of Korea; imcharlotte@anyang.ac.kr

**Keywords:** failure acceptance, entrepreneurial orientation, social safety nets, entrepreneurial intention, entrepreneurial event model

## Abstract

This study aims to examine innovation using an entrepreneurial event model by exploring the effects of failure acceptance, entrepreneurial orientation, and social safety nets on entrepreneurial intention. A survey was conducted with employees in South Korea to collect data, which were analyzed statistically using SPSS version 27.0 and Macro 4.1. The findings indicate that the failure acceptance of South Korean employees does not directly influence their entrepreneurial intention; rather, it has a complete mediation effect through entrepreneurial orientation. This reflects the unique entrepreneurial culture in South Korea, where entrepreneurship is highly emphasized. Additionally, perceptions of social safety nets positively moderate the relationship between entrepreneurial orientation and entrepreneurial intention. The research highlights the significance and direction of studies in the entrepreneurial sector by considering national cultural differences and emphasizing the interaction between individual psychological traits and environmental factors. Furthermore, it presents South Korea’s distinctive entrepreneurial culture and offers elements that could enhance the entrepreneurial environment, thereby creating practical value.

## 1. Introduction

The startup ecosystem in South Korea (Korea hereafter) stands at a significant turning point regarding the perception of failure. In the past, the dominant view was that failure was simply a negative outcome. However, recently, there is a rising movement to break away from this perspective and reinterpret failure as an essential process for growth and innovation. This change signifies a fundamental paradigm shift in startup culture, which has injected new vitality into the Korean startup ecosystem. Notably, evolution of the concept of failure acceptance is remarkable. That is, failure acceptance has transcended mere tolerance for failure and has adopted a more proactive and constructive meaning (Hsu et al., 2017). Specifically, failure acceptance involves recognizing failures encountered during the entrepreneurial process as valuable learning experiences that facilitate personal and organizational growth (Artinger & Powell, 2016). Individuals with high failure acceptance view failures as sources of new ideas, gaining unique insights from unsuccessful projects or concepts that they can leverage to develop more innovative solutions (Larson, 2000). In this context, the role of entrepreneurial orientation is becoming more prominent. In South Korea’s startup environment, entrepreneurial orientation (EO) plays a crucial role in positively influencing the formation of entrepreneurial intention (EI) through failure acceptance. EO enhances individuals’ resilience and adaptability, empowering them to recover quickly from setbacks and seek new alternatives in the face of adversity (Corner et al., 2017).

Korea’s entrepreneurial landscape has achieved quantitative growth; however, there remains room for improvement in terms of qualitative growth and sustainability. According to data from the Ministry of SMEs and Startups (where SME refers to small and medium-sized enterprises; this ministry is a government agency of South Korea established in 2017), technology startups increased by 4.7% in 2020 compared to the previous year; however, they accounted for only 23.3% of all startups. This shows that there is a lack of practical implementation of entrepreneurial spirit in Korean society. In this context, social safety nets are becoming increasingly important. When the culture and institutional environment recognize failure as a learning opportunity and support new challenges, psychological barriers to starting a business are lowered, leading to a surge in innovative startup activities. This perspective’s effectiveness is clearly demonstrated in global leading startup ecosystems. Most companies in Silicon Valley have an average of three failure experiences, and the culture of “fail fast, fail often” has become widely accepted (Engel, 2015). Similarly, despite the high failure rate, the culture in Israel tolerates failure and encourages re-entrepreneurship, functioning as a social safety net. Institutional support also plays a crucial role. Silicon Valley’s “Pay it Forward” system and the Israeli government’s policies supporting entrepreneurs with failure experiences help lower the psychological barriers to their business and strengthen the process through which EO translates into actual startup activities (Ester, 2017; Chosun, 2019).

In this context, the entrepreneurial event model proposed by Shapero and Sokol (1982) provides a theoretical framework for explaining the process of forming EI. This model emphasizes three key elements: perceived desirability, perceived feasibility, and the propensity to act, which serve as useful constructs for understanding the roles of failure acceptance, EO, and social safety nets in this study. First, perceived desirability refers to the perception of the attractiveness of startup; in societies with high failure acceptance, starting a business may be perceived as a more desirable choice and may be viewed as a more desirable choice. Additionally, perceived feasibility reflects the belief in one’s ability to succeed in starting a business, and individuals with strong EO traits are likely to have higher perceived feasibility. Finally, propensity to act signifies the inclination to seize opportunities and take action. A social safety net that allows recovery from failure can further enhance an individual’s propensity to act, facilitating the transition to entrepreneurial behavior. Thus, elements of the entrepreneurial event model define the relationships among the key variables in this study and provide a theoretical basis for presenting the research model.

Meanwhile, research related to entrepreneurship offers valuable insights into EI and its influencing factors from various perspectives. Liu et al. (2011) indicated that individuals with EI may not necessarily translate those intentions into actual startups due to personal characteristics and the surrounding environment, emphasizing the need for research on the gap between intention and action. Barba-Sánchez et al. (2022) highlighted the importance of contextual factors by exploring external environmental influences on the formation of EI. Additionally, He et al. (2020) expanded the understanding of the role of failure by analyzing how past failure experiences impact subsequent EI.

While previous studies have made significant contributions to understanding EI and its influencing factors, there remain several important research gaps that this study aims to address. Most studies have focused on significant elements such as EI and behavior, the external environment, and experiences with failure. However, they largely overlooked the complex interaction between individual entrepreneurial characteristics and social context. While they acknowledged the importance of contextual factors, they did not thoroughly investigate how specific social structures and support mechanisms could influence the relationship between personal characteristics (such as acceptance of failure) and EI. To overcome these limitations, this study proposes a comprehensive approach to examining the factors affecting individuals’ EI in Korean society. Specifically, it investigates the effect of failure acceptance on EI while simultaneously exploring the mediating effect of EO and the moderating effect of social safety nets in this process. This approach extends previous studies by operationalizing the three core elements of the entrepreneurial event model into the specific variables of failure acceptance, EO, and social safety nets, and it comprehensively considers the relational influences among them. This aspect of the study offers a more nuanced understanding of the EI formation process by investigating how broader societal support structures can influence the relationship between individual traits and EI.

The research results are expected to provide a more in-depth understanding of the startup ecosystem in Korean society, suggest customized startup support policy directions that can be applied flexibly to individuals and situations, and ultimately contribute to startup activation and economic innovation.

## 2. Theoretical Background

### 2.1. Social Safety Net

Social safety nets refer to social security systems designed to mitigate various social risks and ensure basic living standards for citizens when they face such risks. These systems enhance individuals’ quality of life, promote overall social welfare, and provide economic and social stability. Social safety nets primarily comprise the income security systems, medical security systems, and social welfare services, with each element working complementarily to form a comprehensive protection system. Social safety net programs help reduce the risk of income loss due to unemployment, retirement, illness, or other factors, thereby promoting individuals’ life stability and reducing social inequality (Wang et al., 2024).

From the economic perspective, social safety nets play a crucial role. In Asian countries, social safety nets are closely linked to economic development (Wang et al., 2024), helping reduce social inequality, distribute economic opportunities equitably, and strengthen social cohesion. In particular, social safety nets are a factor promoting entrepreneurial spirit and startups by mitigating the economic uncertainties faced by entrepreneurs when pursuing innovation. This helps manage the uncertainties entrepreneurs experience in a rapidly changing economic environment and ensures business continuity even in adverse situations such as economic crises and pandemics.

The concept of social safety nets can be extended beyond traditional economic and institutional support to include cultural dimensions. This contributes to forming a culture of social solidarity and mutual support, strengthening community consciousness, and increasing social capital. For example, in communities with diverse cultural backgrounds, it is emphasized that the cultural appropriateness of social safety nets should be considered when addressing health-related issues (Sandesara et al., 2023). In this context, a cultural environment that tolerates failure and recognizes it as an opportunity for learning can be considered an important form of a social safety net.

### 2.2. Failure Acceptance

Failure acceptance refers to an individual’s attitude and ability to perceive and embrace failure, not as something to be feared or avoided but as an opportunity for learning and growth. This includes the ability to derive lessons from failure and the attitude of striving for better results based on these lessons. The concept of failure acceptance has gradually evolved from research on achievement motivation. Early researchers distinguished between the motivations to pursue success and avoid failure. Over time, the perception of failure has changed. While failure was traditionally viewed negatively, recent studies have focused on the instructional aspects of failure.

According to researchers from the University of Chicago, participants who received failure feedback exhibited lower learning effects compared to those who received success feedback. They suggested that this is because failure acts as a threat to the self, causing distraction in learning situations (Eskreis-Winkler & Fishbach, 2019). Therefore, to use failure as a learning opportunity, it is important to have an attitude that accepts failure without perceiving it as a threat. Furthermore, failure acceptance is closely related to the psychological capital theory. Psychological capital consists of elements such as self-efficacy, hope, optimism, and resilience, and these psychological resources enable one to overcome failure and take on new challenges (De Hoe & Janssen, 2022). Carmeli and Hartmann (2024) showed that the learning agility orientation can enhance resilience through the motivation to learn from both success and failure. Additionally, Marangos (2022) emphasized the importance of an open mindset for using failure as a learning opportunity.

The importance of failure acceptance is emphasized not only at the individual level but also in the context of social learning. Research findings showing a higher ability to learn from others’ failures demonstrate that failure acceptance plays an important role in social interactions as well (Kapur, 2015). According to the social learning theory, people learn by observing others’ behaviors and their consequences, and when they see others fail, they have the opportunity to analyze the consequences of that failure and reflect on them in their own behavior. This helps individuals view failure as an opportunity for growth instead of something to fear.

### 2.3. Entrepreneurial Intention

EI is the psychological state of individuals before they actually start a business, and it is regarded as a key predictor of entrepreneurial behavior. The main theoretical models of EI include Ajzen’s (1991) theory of planned behavior and Shapero and Sokol’s (1982) entrepreneurial event model.

According to Ajzen’s (1991) theory of planned behavior, EI is determined by the following three factors: attitude, subjective norms, and perceived behavioral control. Attitude refers to an individual’s positive or negative evaluation of startup, while subjective norms represent the perception of social pressure or expectations regarding startup (Tornikoski & Maalaoui, 2019). Perceived behavioral control, the third component, reflects an individual’s perception of their capability to perform a specific behavior, influenced by both internal factors (e.g., self-efficacy) and external constraints (e.g., resources or opportunities) (La Barbera & Ajzen, 2021; Vamvaka et al., 2020).

The entrepreneurial event model explains the factors influencing the formation of EI. This model emphasizes the following three elements: perceived desirability, perceived feasibility, and the propensity to act. Perceived desirability refers to how attractive startup is to an individual, perceived feasibility represents an individual’s belief in their ability to successfully carry out entrepreneurial activities, and propensity to act refers to an individual’s tendency to seize opportunities and take action (Shapero & Sokol, 1982).

In addition, Bandura’s (1977) social cognitive theory and Schumpeter’s (2003) theory of entrepreneurship provide a theoretical background for the formation of EI. These theories explain that EI is formed through a complex process involving the interaction of various factors such as an individual’s psychological characteristics, social environment, experiences, and education.

### 2.4. Entrepreneurial Orientation

Entrepreneurial spirit is a core characteristic of businesses and entrepreneurs, representing the ability to discover and create new business opportunities, actively pursue these opportunities, and efficiently execute them (Cole, 1959). One of the foundational concepts in understanding entrepreneurial spirit is entrepreneurial orientation (EO). Originating from the work of Miller (1983) and later refined by Covin and Slevin (1988), EO represents a firm’s strategic posture toward entrepreneurial activity. It captures the processes, practices, and decision-making styles that enable organizations to innovate, proactively respond to opportunities, and take calculated risks to achieve competitive advantage. As a unifying framework, EO integrates these behaviors into a measurable construct, providing insights into how individuals and organizations cultivate and sustain entrepreneurial initiatives (Wales et al., 2013; Kreiser & Davis, 2010).

EO is predominantly defined through the following three core dimensions: innovativeness, proactiveness, and risk-taking. Innovativeness reflects a commitment to fostering creativity, experimentation, and the development of new products or services. This dimension emphasizes the importance of novelty and technological advancement as drivers of growth and differentiation (Brettel et al., 2015; Kreiser & Davis, 2010). Proactiveness denotes a forward-looking perspective, characterized by the anticipation of future trends, the identification of market opportunities, and actions taken ahead of competitors. It underscores the importance of ability to lead change rather than react to it (Corrêa et al., 2022; Rank & Strenge, 2018). Risk-taking, the third dimension, refers to the willingness to invest significant resources in projects with uncertain outcomes. This dimension highlights the calculated risks undertaken to achieve potentially transformative results (Brettel et al., 2015).

## 3. Hypothesis Development

### 3.1. Failure Acceptance and Entrepreneurial Orientation

In the field of psychology, an individual’s fear of failure is considered a self-assessment framework that influences how that individual defines and experiences failure in achievement situations (Heckhausen & Heckhausen, 1991). For employees, fear of failure means taking the risk of losing their existing stable standard of living. This fear can weaken the entrepreneurial spirit that seeks to change familiar daily patterns and influence individuals’ achievement motivation, career aspirations, and decision regarding whether to exploit business opportunities (Caliendo et al., 2009; Vaillant & Lafuente, 2007; Welpe et al., 2012). Conversely, in cultures with high failure acceptance, less stigma is attached to failed entrepreneurs and more opportunities are given for second chances, which positively affects the overall cultivation of EO (Dorado, 2005). Some potential entrepreneurs may fear change due to the various risks associated with business failure (Liu et al., 2011). Nevertheless, an attitude of accepting failure can be an important factor in effectively managing and overcoming these fears. Individuals with high failure acceptance can perceive failure as an opportunity for learning and growth, and this attitude and mindset can positively influence the cultivation of EO. The attitude of accepting failure as not something negative but a natural part of the entrepreneurial process contributes to strengthening creativity and the spirit of challenge (Ferrary & Granovetter, 2009). In this context, failure acceptance can act as an important antecedent to EO. Therefore, this study proposes the following hypothesis:

**Hypothesis** **1:** *Failure acceptance positively affects the cultivation of EO*.

### 3.2. Failure Acceptance and Entrepreneurial Intention

How an individual’s psychological characteristics affect EI during the startup process is very important, especially in environments involving uncertainty and risk. In this context, failure acceptance and fear of failure can play a crucial role in shaping EI. In cases of high failure acceptance, the perceived desirability proposed in the entrepreneurial event model (Shapero & Sokol, 1982) may increase, making it more likely for a startup to be perceived as not simply a risky challenge but a valuable experience and opportunity for growth. Tha (2017) showed that a higher fear of failure leads to lower EI. Particularly, fear of shame and embarrassment, lowered self-esteem, and fear of an uncertain future had significant negative relationships with EI. In contrast, individuals who accept failure perceive failure experiences as valuable assets and have an attitude of pursuing new challenges based on these experiences (Artinger & Powell, 2016). This can make potential entrepreneurs evaluate startups as a more attractive career choice, ultimately strengthening EI. Additionally, an attitude of accepting failure can enhance entrepreneurs’ self-efficacy and provide them with the confidence needed to explore new opportunities, thereby strengthening EI. People who do not fear failure recover faster when faced with adversity and seek new directions (Corner et al., 2017). Consequently, individuals with high failure acceptance are likely to have lower psychological barriers to EO and higher EI. Therefore, this study proposes the following hypothesis:

**Hypothesis** **2:** *Failure acceptance positively affects EI.*

### 3.3. Entrepreneurial Orientation and Entrepreneurial Intention

Entrepreneurial orientation (EO) consists of core elements such as innovativeness, risk-taking propensity, and proactiveness, which play crucial roles in the startup process. These characteristics serve as a driving force, providing continuous motivation and confidence to individuals as they navigate the challenges of their business (Huang et al., 2021). Innovativeness enhances one’s ability to identify new ideas and opportunities, providing strong motivation for startups. Risk-taking propensity offers the courage to make decisions and act in uncertain situations, lowering barriers to startups. Proactiveness enables independent and proactive decision-making and action, increasing confidence in the startup process (Shi et al., 2020).

Empirical research highlights that these entrepreneurial traits enhance individuals’ perceptions of their ability to succeed, increasing their intention to engage in entrepreneurial activities (Martins & Perez, 2020; Koe, 2016). Şahin et al. (2019) reported that entrepreneurial self-efficacy and creativity significantly affect EI, suggesting that EO provides a psychological foundation for increasing individual EI. This aligns with the entrepreneurial event model of Shapero and Sokol (1982), which explains how EI is formed. The model presents a close relationship between perceived feasibility, representing an individual’s belief in their ability to succeed in a startup, and EO. Perceived feasibility refers to the degree to which an individual believes they can mobilize the human, social, and financial resources necessary to establish a new venture, and this is closely related to confidence in their own startup abilities. Therefore, this study proposes the following hypothesis:

**Hypothesis** **3:** *EO positively affects EI.*

### 3.4. Mediating Effect of Entrepreneurial Orientation on the Relationship Between Failure Acceptance and Entrepreneurial Intention

EI is an important psychological factor that drives entrepreneurial behavior, explaining entrepreneurs’ willingness and motivation to start a new business. Cheng et al. (2024) explored how internal expectations and failure experiences influence entrepreneurial choices, suggesting that failure acceptance provides an important psychological foundation for strengthening EI. Various psychological and personal factors play a crucial role in forming EI, and how entrepreneurs perceive and accept failure can have a significant impact. In the startup process, failure is often considered an inevitable experience, and acceptance of such failure refers to the attitude and ability to perceive it as a positive learning opportunity and a chance for growth. This attitude and ability can enhance EO based on the perceived feasibility of new ventures. From this perspective, recent studies suggest that failure acceptance can promote an entrepreneur’s spirit and ultimately help strengthen EI. Hsu et al. (2017) showed that while failure experiences can temporarily weaken self-efficacy, individuals with high failure acceptance can recover their confidence and attempt new challenges. Corner et al. (2017) demonstrated that entrepreneurs who have experienced failure can develop better strategies and attempt new challenges through resilience. This provides additional evidence explaining the process by which failure acceptance promotes the development of EO and consequently strengthens EI. Therefore, this study proposes the following hypothesis:

**Hypothesis** **4:** *EO mediates the relationship between failure acceptance and EI.*

### 3.5. Moderating Effect of Social Safety Net on the Relationship Between Entrepreneurial Orientation and Entrepreneurial Intention

EO has been studied extensively as a crucial factor in shaping EI. However, the risk of failure is always present when starting a business, which instills fear in potential entrepreneurs. In this context, the role of social safety nets becomes extremely important. Social safety nets provide a cushion against potential adverse outcomes. By mitigating personal and economic risks, social safety nets can alleviate the fear associated with starting a business, making individuals more likely to act on their intentions. When individuals perceive robust social safety nets, they are more likely to view their business as feasible and less threatening, thereby amplifying the positive influence of EO on EI. Acs et al. (2008) verified that improvements in bankruptcy laws increased startup activity, suggesting that institutional mechanisms that mitigate the negative consequences of business failure can enhance entrepreneurial will. Estrin et al. (2013) demonstrated that social capital can increase the likelihood of an individual entering a commercial startup. Additionally, Maclean et al. (2013) argued that factors such as social support can play a crucial role in strengthening EI. These research findings suggest that the psychological stability provided by social safety nets can reduce the fear of starting a business and, consequently, strengthen the effect of EO leading to EI. Therefore, this study proposes the following hypothesis:

**Hypothesis** **5:** *A positive perception of social safety nets has a moderating effect that strengthens the relationship between EO and EI.*

**Figure 1:**
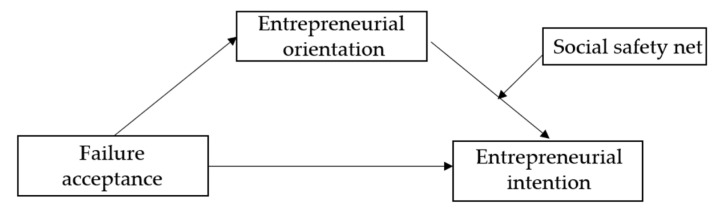
Research model.

Figure 1 presents the research model summarizing these hypotheses.

## 4. Research Methods

### 4.1. Data Analysis

This study’s sample consisted of individuals aged 20 years and above who are employed in Korea. The research was conducted through an online survey over a two-month period from July to September 2023. A total of 286 questionnaires were collected, and 282 surveys were used for the final analysis after excluding four that had many missing values or insincere responses. Table 1 presents the demographic characteristics of the research subjects.

Statistical analysis was performed using SPSS 27.0 and Macro 4.1 software, and the specific analysis methods are as follows. First, frequency analysis was conducted to identify the research participants’ demographic characteristics. Next, an exploratory factor analysis was performed to evaluate the validity of the measurement tools, and Cronbach’s alpha coefficients were calculated to examine the reliability of the measurement items. Descriptive statistical analysis and Pearson’s correlation analysis were conducted to confirm the levels of major variables and analyze the correlations between the variables. Additionally, confirmatory factor analysis was performed to verify convergent and discriminant validity, and factor loadings, average variance extracted (AVE), and concept reliability were calculated. For the mediation effect test, 2000 bootstrapping iterations were used, and multiple regression analysis was applied for the moderation effect test. All statistical significance was judged at a significance level of 0.05.

### 4.2. Questionnaire Composition

Table 2 presents the questionnaire composition for this study. To measure failure acceptance, this study followed the methods of Lang and Fries (2006) and Boyd and Gumpert (1983) to measure the fear of failure, which was reverse-scored. To measure the EO, we adopted the three dimensions of innovation, risk-taking, and proactiveness suggested by Miller (1983), and modified and used the scale of Covin and Slevin (1988), which is currently the most widely used in entrepreneurship research. In addition, the items used by Lee (2016) and An and Chung (2021) were used to measure awareness of the social safety net, and the measurement tool developed and used by Crant (1996) was used to measure EI.

### 4.3. Validity and Reliability of the Measurement Tool

An exploratory factor analysis was performed to evaluate the factor structure and validity of the measurement tool. Principal component analysis was applied as a factor extraction method, and varimax, a type of orthogonal rotation, was used as a factor rotation method. If the factor loading of an item was 0.4 or higher, it was judged to belong to the corresponding factor (Ford et al., 1986), and the reliability of the variables derived through factor analysis was evaluated by the items’ internal fit coefficient. A coefficient being 0.6 or higher was interpreted as acceptably reliable (Nunnally, 1978).

The exploratory factor analysis indicated that item six of EO did not meet the minimum factor loading criteria; thus, this item was removed, and the exploratory factor analysis was conducted again. Table 3 presents the final analysis results. The Kaiser–Meyer–Olkin measure, which determines the adequacy of the sample size, was 0.800, which is higher than the minimum standard of 0.6; moreover, Bartlett’s test of sphericity, which determines whether the matrix is a unit matrix, was significant with the 0.05 significance level. This confirmed that the collected data were suitable for the exploratory factor analysis.

Six factors with eigenvalues greater than 1.0 were extracted, and each factor’s explanatory power was as follows: failure acceptance, 12.34%; innovativeness, 15.30%; risk-taking propensity, 8.82%; proactiveness, 10.87%; perception of social safety net, 14.57%; and EI, 14.85%. All items showed factor loadings greater than 0.4, and the internal consistency coefficients (Cronbach’s alpha) were 0.77 for failure acceptance, 0.81 for innovativeness, 0.74 for risk-taking propensity, 0.73 for proactiveness, 0.89 for perception of social safety net, and 0.89 for EI, confirming good validity and reliability of the measurement tool.

### 4.4. Descriptive Statistics and Correlation Analysis

Table 4 presents the results of the descriptive statistics and correlations of the main variables in this study. To verify the general tendencies of the main variables and the assumption of normality, we calculated the mean and standard deviation, as well as checked the skewness and kurtosis values. The variables’ means were as follows: failure acceptance, 2.84; EO, 3.52; innovativeness, 3.60; risk taking, 3.60; proactiveness, 2.98; social safety net, 2.62; and EI, 3.17. The range of skewness for the variables was from −0.57 to 0.16, and the range of kurtosis was from −0.62 to 1.04. As the skewness was less than 3 and kurtosis less than 7, we confirmed that all variables satisfied the assumption of normal distribution (Kline, 2023).

Furthermore, Pearson’s correlation analysis examined the relationships between the main variables, showing that failure acceptance had positive correlations with EO (r = 0.23, *p* < 0.001), innovativeness (r = 0.12, *p* < 0.05), risk taking (r = 0.22, *p* < 0.001), proactiveness (r = 0.23, *p* < 0.001), and social safety net (r = 0.13, *p* < 0.05). Moreover, EO showed a positive correlation with EI (r = 0.59, *p* < 0.001). However, the relationships between failure acceptance and EI, between EO and perception of social safety net, and between social safety net and EI were not significant. Additionally, we confirmed that there was no multicollinearity issue, as the variance inflation factor values for failure acceptance, EO, and social safety net were all below 10.

### 4.5. Measurement Model Testing

To verify the measurement model’s convergent and discriminant validity, we conducted a confirmatory factor analysis. To assess the model’s fit, we examined the chi-square test statistic, incremental fit index (IFI), Tucker–Lewis index (TLI), comparative fit index (CFI), and root mean square error of approximation (RMSEA). The minimum criteria for acceptable model fit are IFI, TLI, and CFI values above 0.9 and RMSEA below 0.10 (Bentler, 1990; Browne & Cudeck, 1992). The model fit indices were IFI = 0.965, TLI = 0.951, CFI = 0.964, and RMSEA = 0.065, indicating an acceptable model fit.

Table 5 presents the standardized regression coefficients, AVE, and construct reliability (CR). Convergent validity is considered satisfactory when the standardized regression coefficients and AVE are above 0.5 and CR is above 0.7 (Anderson & Gerbing, 1988). In this study, the standardized regression coefficients ranged from 0.61 to 0.92, AVE from 0.52 to 0.73, and CR from 0.77 to 0.89, confirming that convergent validity was established.

Discriminant validity is verified using the HTMT (heterotrait/monotrait ratio of correlations) method. In HTMT, the correlation between two different latent variables is evaluated. For this purpose, the correlations between items or sub-factors belonging to each latent variable are calculated, and the relationship between the two latent variables is measured through the ratio of these correlations. In this method, discriminant validity is considered satisfactory if the HTMT between latent variables is less than 0.85 (Henseler et al., 2015). As shown in Table 6, the HTMT among all latent variables is less than 0.85, confirming that discriminant validity is proven.

### 4.6. Hypothesis Testing

#### 4.6.1. Verification of the Mediating Effect of Entrepreneurial Orientation

To test the mediating effect of EO on the relationship between failure acceptance and EI, we conducted an analysis using Macro Model 4 (Hayes, 2015). Gender, age, education level, employment contract type, and tenure were set as control variables. Table 7 presents the results of the analysis.

In the direct effects, gender (B = 0.23, confidence interval [CI] = 0.07~0.38), education level (B = 0.41, CI = 0.20~0.62), employment contract type (B = 0.28, CI = 0.06~0.51), and failure acceptance (B = 0.20, CI = 0.11~0.30) showed positive effects on EO. Specifically, men compared to women, those with a bachelor’s degree or higher compared to those without, full-time employees compared to contract workers, and those with higher compared to lower failure acceptance showed higher levels of EO. Therefore, Hypothesis 1, which posited that failure acceptance would have a positive effect on EO, was supported.

Additionally, gender (B = 0.36, CI = 0.14~0.58), education level (B = 0.44, CI = 0.14~0.75), tenure (B = 0.27, CI = 0.06~0.48), and EO (B = 0.93, CI = 0.77~1.10) showed positive effects on EI, while failure acceptance did not have a significant effect. This indicates that men compared to women, those with a bachelor’s degree or higher compared to those without, those with five or more years of tenure compared to those with less, and those with higher EO showed higher EI. Consequently, Hypothesis 3, which stated that EO would have a positive effect on EI, was supported, while Hypothesis 2, which proposed that failure acceptance would have a positive effect on EI, was rejected.

Regarding the indirect effects, the indirect path from failure acceptance to EI through EO was found to be significant (B = 0.19, CI = 0.09~0.29). This indicates a full mediation effect, where failure acceptance does not influence EI directly but only indirectly through EO. Therefore, Hypothesis 4, which posited that EO would mediate the relationship between failure acceptance and EI, was supported.

#### 4.6.2. Verification of the Moderating Effect of the Social Safety Net

To test the moderating effect of the social safety net on the relationship between EO and EI, we conducted an analysis using Macro Model 1 (Hayes, 2015). Gender, age, education level, employment contract type, and tenure were set as control variables. To avoid multicollinearity issues, the independent variable and moderator variable were mean-centered. Table 8 presents the results.

The analysis results showed that the interaction term between EO and perception of social safety net (B = 0.13, CI = 0.04~0.22) was significant. This confirms that the perception of social safety net moderates the relationship between EO and EI. Therefore, Hypothesis 5, which posited that the perception of social safety net moderates the relationship between EO and EI, was supported.

**Figure 2:**
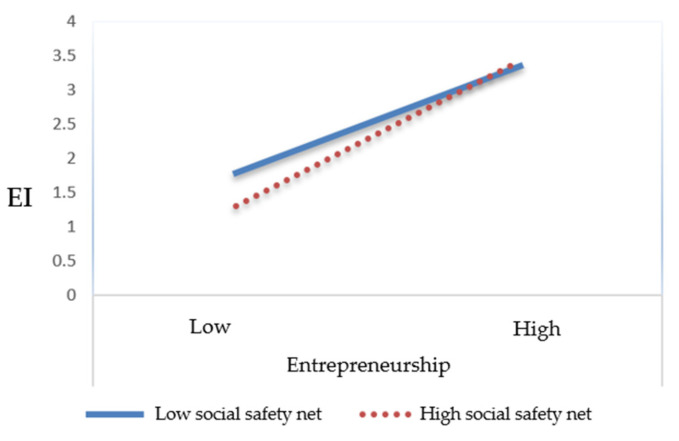
Moderating effect of the social safety net. EI: entrepreneurial intention.

A simple slope analysis was conducted to determine the significance of the effect of EO on EI according to the level of the social safety net. Specifically, the effect of EO on EI was estimated at points ±1 standard deviation from the mean of the social safety net. As shown in Table 9 and Figure 2, when the perception of the social safety net was high, the increase in EI was greater as EO increased. Conversely, when the perception of the social safety net was low, the degree to which EI increased as EO increased was relatively lower.

## 5. Conclusions

### 5.1. Key Findings

The main results and discussions of this study are as follows.

First, failure acceptance was found to have a positive (+) effect on EO, supporting Hypothesis 1 and confirming previous research (Ferrary & Granovetter, 2009). This finding suggests that entrepreneurs with high failure acceptance maintain a positive attitude toward overcoming failure and exploring new opportunities rather than fearing it. As a result, the overall level of EO among founders is enhanced, which serves to further strengthen the motivation and capabilities for entrepreneurial activities.

Second, EO was confirmed to have a positive (+) effect on EI. This supports Hypothesis 3 and aligns with the research findings (Huang et al., 2021; Shi et al., 2020). Individuals with high EO actively pursue creative and innovative ideas and have a stronger tendency to recognize business opportunities and put them into practice. In particular, entrepreneurs with high EO clearly demonstrate EI by taking risks and challenging new markets. This is an important result confirming that EO is a key factor in stimulating EI.

Third, failure acceptance was found to have a full mediating effect on EI through EO, supporting Hypothesis 4. In other words, the attitude of accepting failure alone does not increase EI directly but does so indirectly by strengthening EO. This indicates a complex process where the attitude of accepting failure as a positive experience enhances the entrepreneur’s spirit of challenge and creativity, ultimately leading to EI. These results provide an important implication that failure acceptance plays a crucial role in the entrepreneurial process, but its influence is manifested through the medium of EO.

Fourth, the social safety net was found to moderate the relationship between EO and EI, supporting Hypothesis 5. This suggests that when there is a social safety net that can minimize losses from entrepreneurial failure, the positive attitude towards EO is further enhanced. Thus, the existence of a social safety net plays an important role in providing psychological stability, allowing entrepreneurs to take more risks and attempt EO.

### 5.2. Implications

#### 5.2.1. Academic Implications

The academic implications are as follows.

First, this study identified the full mediating effect of EO in the relationship between failure acceptance and EI. It revealed that failure acceptance does not influence EI directly but indirectly through EO. This clarified the mechanism by which failure acceptance strengthens EO, and enhanced EO increases EI. Specifically, this study empirically verified the process by which the attitude of not fearing failure reinforces entrepreneurial characteristics such as innovativeness, risk taking, and proactiveness, which in turn lead to increased EI. These findings provide a more sophisticated understanding of the EI formation process and emphasize the importance of failure acceptance.

Second, this study has academic significance as it expanded and concretized Shapero and Sokol’s (1982) entrepreneurial event model, which explains the EI formation process, to fit the context of this research. The abstract concepts in the existing model, such as “perceived desirability”, “perceived feasibility”, and “propensity to act”, were operationalized into measurable variables such as failure acceptance, EO, and perception of social safety nets. This expanded the practical applicability of the existing theory and strengthened the theoretical foundation of entrepreneurship research.

Third, the study verified the impact of the interaction between individual psychological characteristics (EO) and environmental factors (social safety net) on EI. These results emphasized the importance of an integrated consideration of individuals and the environment in entrepreneurship research, and they provided a new perspective on the role of social safety nets in creating an entrepreneurial ecosystem.

#### 5.2.2. Practical Implications

The practical implications of this study’s results are as follows.

The fact that Korean workers’ tolerance for failure does not affect their intention to start a business directly but rather indirectly through their entrepreneurial spirit reflects the country’s unique entrepreneurial culture. It suggests that Korea’s economic growth has been achieved through bold challenges based on the entrepreneurial spirit of outstanding entrepreneurs. However, this entrepreneurial spirit has been gradually weakening in the Korean economy lately, which may weaken the spirit of challenge for starting a business and hinder economic growth. It seems that the anti-business sentiment prevalent throughout society, the fear of failure, and a culture of preferring stable jobs are discouraging the young generation’s will to start a business. In addition, factors that hinder business activities, such as excessive regulations and high tax burdens, are contributing to the weakening of entrepreneurial spirit. Therefore, various activities and efforts at the corporate, university, and national levels are necessary to strengthen the entrepreneurial spirit. Specifically, companies should activate internal entrepreneurship programs and support employees with innovative ideas. In particular, large corporations can foster their employees’ creativity and spirit of challenge through in-house venture systems. Universities need to strengthen entrepreneurship education and expand programs that provide students with real-world entrepreneurial experiences. For example, we can encourage students’ entrepreneurial spirit by activating startup clubs, hosting startup competitions, and operating incubation centers. Finally, the government should expand startup support policies and ease regulations to activate the startup ecosystem. We can reduce the burden of startups and encourage the spirit of challenge by providing tax benefits, startup funding support, and opportunities for re-challenge in case of failure. Through these multifaceted efforts, we can activate Korea’s startup ecosystem and maintain the momentum of economic growth.

### 5.3. Limitations and Future Research

The limitations of this study are as follows.

First, this study is based on a cross-sectional analysis using data collected at a single time point. This approach is limited in capturing the dynamic relationships and changes over time between failure acceptance, EO, and EI, thus limiting the ability to clearly identify causal relationships between variables. Future research needs to overcome this limitation through longitudinal research designs using time-series data. For example, regular data collection over a certain period for a specific group can be conducted to observe the changing trends in the formation of EI. This will allow for a more accurate understanding of the variability of each variable over time, the dynamic patterns of interaction between variables, and long-term causal relationships.

Second, this study was conducted within a specific cultural context and did not fully consider the impact of cultural differences on the results. Future research must explore the influence of the cultural context on the process of EI formation through comparative studies in various cultural settings.

Third, various external factors that could influence EI were not fully controlled for in this study. In particular, economic conditions, changes in government policies, and industry trends can significantly impact an individual’s entrepreneurial decision-making process. Therefore, thoroughly incorporating these factors remains a challenge for future research.

## Figures and Tables

**Table 1:** Demographic characteristics.

Variables		*N =* 288	%
Gender	Men	171	60.6
	Women	111	39.4
Age (years)	20s	68	24.6
	30s	113	40.1
	40s	45	16.0
	50s	42	14.9
	60s and above	70	24.8
Education	High school graduate or lower	30	10.6
	University graduate	200	70.9
	Graduate school graduate	52	18.4
Marital status	Single	81	28.7
	Married	190	67.4
	Other (widowed, separated, etc.)	11	3.9
Average monthly income	<KRW 2 million	4	1.4
	KRW 2–3 million	46	16.3
	KRW 3–4 million	73	25.9
	KRW 4–5 million	36	12.8
	KRW 5–6 million	38	13.5
	≥KRW 6 million	85	30.1
Types of employment contracts	Full-time	242	85.8
	Contractual	40	14.2
Tenure	<2 years	39	13.8
	2–5 years	90	31.9
	5–10 years	78	27.7
	10–20 years	56	19.9
	≥20 years	19	6.7
Area of residence	Metropolitan area	212	75.2
	Non-metropolitan area	70	24.8

**Table 2:** Questionnaire composition.

Variable		Operational Definition	Items
Failure acceptance		This is measured by reverse-scoring the Fear of Failure Scale. This scale assesses the degree of psychological stress, pressure, and negative emotions experienced in a situation of failure. A high reverse score indicates low fear of failure and high ability to accept failure, while a low score indicates the opposite.	When more depends on me in difficult situations, I tend to fear failure.
			I tend to feel uncomfortable if I don’t have confidence in succeeding at what I’m doing.
			Even if others are not watching, I tend to feel anxious when facing new situations.
Entrepreneurial orientation	Innovativeness	This is the tendency to pursue continuous new product and service development, production technology improvement, and general technology improvement.	I am creative and original.
			I am interested in developing new solutions to problems.
			I look for opportunities to introduce solutions that make my work more efficient.
	Risk-taking	This is the tendency to prefer high-risk projects and the willingness to actively seek and pursue opportunities.	I am creative and original.
			I am interested in developing new solutions to problems.
			I look for opportunities to introduce solutions that make my work more efficient.
	Proactiveness	This refers to an active competitive spirit, the will to create superior performance, and a challenging attitude toward competitors.	I always want to do better than others in whatever I do.
			I want to solve problems, even if they are difficult.
			I can change anything if I put my mind to it.
Social safety net		This refers to an individual’s understanding and evaluation of institutional mechanisms that support the individual’s economic and social recovery in the event of business failure.	Our society provides sufficient support for entrepreneurs to recover when their business fails.
			Our society has a well-established atmosphere and culture in which business failure is not considered shameful.
			Our society has a well-established atmosphere in which business failure experience can become a social asset.
Entrepreneurial intention		This refers to a person’s conscious state of mind and plan to start a new business in the future.	I have always dreamed of starting and running my own business.
			I would be willing to start a business if I had the chance.
			I will start or acquire and run my own business in the relatively near future.

**Table 3:** Validity and reliability of the measurement tools.

Factor	Items	Factor Loading	Eigenvalue	Explained Variance (%)	Cronbach’s α
Innovativeness	Innovativeness 2	0.814	2.60	15.30	0.81
	Innovativeness 1	0.782			
	Innovativeness 3	0.726			
EI	EI 2	0.874	2.52	14.85	0.89
	EI 3	0.857			
	EI 1	0.809			
Social safety net	Social safety net 3	0.922	2.48	14.57	0.89
	Social safety net 2	0.917			
	Social safety net 1	0.852			
Failure acceptance	Failure acceptance 1	0.851	2.10	12.34	0.77
	Failure acceptance 3	0.839			
	Failure acceptance 2	0.771			
Proactiveness	Proactiveness 1	0.814	1.85	10.87	0.73
	Proactiveness 2	0.784			
	Proactiveness 3	0.410			
Risk taking	Risk taking 2	0.622	1.50	8.82	0.74
	Risk taking 1	0.549			
Total				76.74	-
KMO		0.800			
Bartlett’s test of sphericity		*x*^2^(136) = 2630.20 (0.000)			

EI: entrepreneurial intention; KMO: Kaiser–Meyer–Olkin test.

**Table 4:** Descriptive statistics and correlation analysis.

Variable	Mean	SD	Skewness	Kurtosis	1	2	2-1	2-2	2-3	3
1. Failure acceptance	2.84	0.72	0.03	−0.45	1					
2. Entrepreneurial orientation	3.52	0.6	−0.01	−0.09	0.23 ***	1				
2-1. Innovativeness	3.6	0.69	0.05	−0.05	0.12 *	0.85 ***	1			
2-2. Risk taking	2.98	0.88	−0.07	−0.52	0.22 ***	0.82 ***	0.59 ***	1		
2-3. Proactiveness	3.81	0.67	−0.57	1.04	0.23 ***	0.81 ***	0.49 ***	0.49 ***	1	
3. Social safety net	2.62	0.85	0.16	0.04	0.13 *	0.10	0.05	0.09	0.11	1
4. Entrepreneurial intention	3.17	0.99	−0.26	−0.62	0.07	0.59 ***	0.53 ***	0.53 ***	0.41 ***	−0.01
VIF					1.08	-	1.67	1.7	1.48	1.03

* *p* < 0.05, *** *p* < 0.001. SD: standard deviation; VIF: variance inflation factor.

**Table 5:** Standardized regression coefficients, average variance extracted (AVE), and construct reliability (CR).

Latent Variable	Measurement Variable	*β*	AVE	CR
Failure acceptance	Failure acceptance 1	0.83	0.55	0.78
	Failure acceptance 2	0.61		
	Failure acceptance 3	0.77		
Entrepreneurial orientation	Innovativeness	0.75	0.52	0.77
	Risk taking	0.77		
	Proactiveness	0.64		
Social safety net	Social safety net 1	0.73	0.73	0.89
	Social safety net 2	0.90		
	Social safety net 3	0.92		
Entrepreneurial intention (EI)	EI 1	0.89	0.71	0.88
	EI 2	0.84		
	EI 3	0.80		

**Table 6:** HTML between latent variables.

	Failure Acceptance	Entrepreneurial Orientation (EO)	Social Safety Net
Failure acceptance			
EO	0.33		
Social safety net	0.15	0.16	
EI	0.08	0.72	0.00

**Table 7:** Mediation effect.

Path		B	SE	*t*	*p*	95% CI	
						LLCI	ULCI
Direct effect	Failure acceptance → EO (H1, supported)	0.20	0.05	4.40	<0.001	0.11	0.30
	Gender (ref: women) → EO	0.23	0.08	2.89	0.004	0.07	0.38
	Age (ref: under 40 years) → EO	0.12	0.08	1.53	0.127	−0.03	0.27
	Education (ref: less than university graduate) → EO	0.41	0.11	3.79	<0.001	0.20	0.62
	Types of employment contracts (ref: contractual) → EO	0.28	0.12	2.45	0.015	0.06	0.51
	Tenure (ref: less than five years) → EO	−0.04	0.08	−0.57	0.569	−0.19	0.11
	Failure acceptance → EI (H2, supported)	−0.12	0.07	−1.74	0.082	−0.25	0.02
	EO → EI (H3, supported)	0.93	0.08	11.10	<0.001	0.77	1.10
	Gender (ref: women) → EI	0.36	0.11	3.25	0.001	0.14	0.58
	Age (ref: under 40 years) → EI	−0.15	0.11	−1.41	0.160	−0.37	0.06
	Education (ref: less than university graduate) → EI	0.44	0.16	2.85	0.005	0.14	0.75
	Types of employment contracts (ref: contractual) → EI	−0.02	0.16	−0.14	0.888	−0.34	0.30
	Tenure (ref: less than five years) → EI	0.27	0.11	2.52	0.012	0.06	0.48
Indirect effect	Failure acceptance → EO → EI (H4, supported)	0.19	0.05	-	-	0.09	0.29

SE: standard error; CI: confidence interval; LLCI: lower limit confidence interval; ULCI: upper limit confidence interval; EI: entrepreneurial intention.

**Table 8:** Moderating effect.

Variable	B	SE	*t*	*p*	95% CI		*F*	R^2^
					LLCI	ULCI		
Entrepreneurial orientation	0.92	0.08	11.13	<0.001	0.76	1.08	28.47 ***	0.428
Social safety net	−0.10	0.06	−1.68	0.095	−0.22	0.02		
Entrepreneurial orientation × Social safety net (H5, supported)	0.13	0.05	2.00	0.048	0.04	0.22		

*** *p* < 0.001. Control variables are omitted. SE: standard error; CI: confidence interval; LLCI: lower limit confidence interval; ULCI: upper limit confidence interval.

**Table 9:** Results of the simple slope analysis.

Moderator		B	SE	*t*	*p*
Social safety net	M − 1SD	0.83	0.11	7.40 ***	<0.001
	M	0.92	0.08	11.13 ***	<0.001
	M + 1SD	1.01	0.12	8.33 ***	<0.001

*** *p* < 0.001. SE: standard error; M: mean; SD: standard deviation.

## Data Availability

Data are contained within the article. Further inquiries can be directed to the corresponding author.

## References

[B1-behavsci-15-00028] Acs Z. J., Desai S., Hessels J. (2008). Entrepreneurship, economic development and institutions. Small Business Economics.

[B2-behavsci-15-00028] Ajzen I. (1991). The theory of planned behavior.

[B3-behavsci-15-00028] An S. -J., Chung J. -H. (2021). Effect of Pressure on Business Failure and Social Safety Network on the Food Service Re-Entrepreneurial Intention: Focusing on Shapero’s Entrepreneurial Event Model. Korean Hospitality and Tourism Academe.

[B4-behavsci-15-00028] Anderson J. C., Gerbing D. W. (1988). Structural equation modeling in practice: A review and recommended two-step approach. Psychological Bulletin.

[B5-behavsci-15-00028] Artinger S., Powell T. C. (2016). Entrepreneurial failure: Statistical and psychological explanations. Strategic Management Journal.

[B6-behavsci-15-00028] Bandura A. (1977). Social learning theory.

[B7-behavsci-15-00028] Barba-Sánchez V., Mitre-Aranda M., del Brío-González J. (2022). The entrepreneurial intention of university students: An environmental perspective. European Research on Management and Business Economics.

[B8-behavsci-15-00028] Bentler P. M. (1990). Comparative fit indexes in structural models. Psychological Bulletin.

[B9-behavsci-15-00028] Boyd D. P., Gumpert D. E. (1983). Coping with entrepreneurial stress. Harvard Business Review.

[B10-behavsci-15-00028] Brettel M., Chomik C., Flatten T. C. (2015). How organizational culture influences innovativeness, proactiveness, and risk-taking: Fostering entrepreneurial orientation in SMEs. Journal of Small Business Management.

[B11-behavsci-15-00028] Browne M. W., Cudeck R. (1992). Alternative ways of assessing model fit. Sociological Methods & Research.

[B12-behavsci-15-00028] Caliendo M., Fossen F. M., Kritikos A. S. (2009). Risk attitudes of nascent entrepreneurs–new evidence from an experimentally validated survey. Small Business Economics.

[B13-behavsci-15-00028] Carmeli A., Hartmann S. (2024). Learning Agility Orientation, Ambidextrous Learning, and Resilience. IEEE Transactions on Engineering Management.

[B14-behavsci-15-00028] Cheng Y., Zheng Y., Schiavone F., Escobar O. R. (2024). Fantasy of success, fear of failure and entrepreneurial choice: The moderating role of business vibrancy and failure experience. International Journal of Entrepreneurial Behavior & Research.

[B15-behavsci-15-00028] Chosun I. (2019). Thanks to the sincere acceptance of failure… Israel is the ‘kingdom’ of startups.

[B16-behavsci-15-00028] Cole A. H. (1959). Business enterprise in its social setting.

[B17-behavsci-15-00028] Corner P. D., Singh S., Pavlovich K. (2017). Entrepreneurial resilience and venture failure. International Small Business Journal.

[B18-behavsci-15-00028] Corrêa V. S., Queiroz M. M., Cruz M. A., Shigaki H. B. (2022). Entrepreneurial orientation far beyond opportunity: The influence of the necessity for innovativeness, proactiveness and risk-taking. International Journal of Entrepreneurial Behavior & Research.

[B19-behavsci-15-00028] Covin J. G., Slevin D. P. (1988). The influence of organization structure on the utility of an entrepreneurial top management style. Journal of Management Studies.

[B20-behavsci-15-00028] Crant J. M. (1996). The proactive personality scale as a predictor of entrepreneurial intentions. Management.

[B21-behavsci-15-00028] De Hoe R., Janssen F. (2022). Re-creation after business failure: A conceptual model of the mediating role of psychological capital. Frontiers in Psychology.

[B22-behavsci-15-00028] Dorado S. (2005). Institutional entrepreneurship, partaking, and convening. Organization Studies.

[B23-behavsci-15-00028] Engel J. S. (2015). Global clusters of innovation: Lessons from Silicon Valley. California Management Review.

[B24-behavsci-15-00028] Eskreis-Winkler L., Fishbach A. (2019). Not learning from failure—The greatest failure of all. Psychological Science.

[B25-behavsci-15-00028] Ester P. (2017). Accelerators in Silicon Valley: Building successful startups.

[B26-behavsci-15-00028] Estrin S., Mickiewicz T., Stephan U. (2013). Entrepreneurship, social capital, and institutions: Social and commercial entrepreneurship across nations. Entrepreneurship Theory and Practice.

[B27-behavsci-15-00028] Ferrary M., Granovetter M. (2009). The role of venture capital firms in Silicon Valley’s complex innovation network. Economy and Society.

[B28-behavsci-15-00028] Ford J. K., MacCallum R. C., Tait M. (1986). The application of exploratory factor analysis in applied psychology: A critical review and analysis. Personnel Psychology.

[B29-behavsci-15-00028] Hayes A. F. (2015). An index and test of linear moderated mediation. Multivariate Behavioral Research.

[B30-behavsci-15-00028] He H., Bai Y., Xiao X. (2020). How past failure predicts subsequent entrepreneurial intention: A comparative study of mainland China and Taiwan. Sustainability.

[B31-behavsci-15-00028] Heckhausen J., Heckhausen H. (1991). Motivation and action.

[B32-behavsci-15-00028] Henseler J., Ringle C. M., Sarstedt M. (2015). A new criterion for assessing discriminant validity in variance-based structural equation modeling. Journal of the Academy of Marketing Science 43.

[B33-behavsci-15-00028] Hsu D. K., Wiklund J., Cotton R. D. (2017). Success, failure, and entrepreneurial reentry: An experimental assessment of the veracity of self–efficacy and prospect theory. Entrepreneurship Theory and Practice.

[B34-behavsci-15-00028] Huang Y., An L., Wang J., Chen Y., Wang S., Wang P. (2021). The role of entrepreneurship policy in college students’ entrepreneurial intention: The intermediary role of entrepreneurial practice and entrepreneurial spirit. Frontiers in Psychology.

[B35-behavsci-15-00028] Kapur M. (2015). Learning from productive failure. Learning: Research and Practice.

[B36-behavsci-15-00028] Kline R. B. (2023). Principles and practice of structural equation modeling.

[B37-behavsci-15-00028] Koe W. L. (2016). The relationship between Individual Entrepreneurial Orientation (IEO) and entrepreneurial intention. Journal of Global Entrepreneurship Research.

[B38-behavsci-15-00028] Kreiser P. M., Davis J. (2010). Entrepreneurial orientation and firm performance: The unique impact of innovativeness, proactiveness, and risk-taking. Journal of Small Business & Entrepreneurship.

[B39-behavsci-15-00028] La Barbera F., Ajzen I. (2021). Moderating role of perceived behavioral control in the theory of planned behavior: A preregistered study. Journal of Theoretical Social Psychology.

[B40-behavsci-15-00028] Lang J. W., Fries S. (2006). A revised 10-item version of the Achievement Motives Scale. European Journal of Psychological Assessment.

[B41-behavsci-15-00028] Larson A. L. (2000). Sustainable innovation through an entrepreneurship lens. Business strategy and the Environment.

[B42-behavsci-15-00028] Lee J. (2016). The motivation for establishing start-ups and the pressure of failure in effecting the will to establish business start-up: A focus on the moderating effect of the social safety network through international comparison. Ph.D. thesis.

[B43-behavsci-15-00028] Liu W., Pei H., Kunpeng X. (2011). Can entrepreneurial opportunities really help entrepreneurship intention? A study based on mixing effect of entrepreneurship self-efficacy and perceived risk. Nankai Business Review.

[B44-behavsci-15-00028] Maclean M., Harvey C., Gordon J. (2013). Social innovation, social entrepreneurship and the practice of contemporary entrepreneurial philanthropy. International Small Business Journal.

[B45-behavsci-15-00028] Marangos D. (2022). Dentistry fails: How failure leads to success. Cranio.

[B46-behavsci-15-00028] Martins I., Perez J. P. (2020). Testing mediating effects of individual entrepreneurial orientation on the relation between close environmental factors and entrepreneurial intention. International Journal of Entrepreneurial Behavior & Research.

[B47-behavsci-15-00028] Miller D. (1983). The correlates of entrepreneurship in three types of firms. Management Science.

[B48-behavsci-15-00028] Nunnally J. C. (1978). An overview of psychological measurement. Clinical diagnosis of mental disorders: A handbook.

[B49-behavsci-15-00028] Rank O. N., Strenge M. (2018). Entrepreneurial orientation as a driver of brokerage in external networks: Exploring the effects of risk taking, proactivity, and innovativeness. Strategic Entrepreneurship Journal.

[B50-behavsci-15-00028] Şahin F., Karadağ H., Tuncer B. (2019). Big five personality traits, entrepreneurial self-efficacy and entrepreneurial intention: A configurational approach. International Journal of Entrepreneurial Behavior & Research.

[B51-behavsci-15-00028] Sandesara U. N., Carson S. L., Dopp A., Perez L. G., Sadia A., Wali S., Park N. J., Casillas A., Kim G., Morales M. G. (2023). Community and healthcare perspectives on implementing hypertension interventions for a multiethnic safety-net population. Ethnicity and Disease.

[B52-behavsci-15-00028] Schumpeter J. (2003). The theory of economic development.

[B53-behavsci-15-00028] Shapero A., Sokol L. (1982). The social dimensions of entrepreneurship.

[B54-behavsci-15-00028] Shi Y., Yuan T., Bell R., Wang J. (2020). Investigating the relationship between creativity and entrepreneurial intention: The moderating role of creativity in the theory of planned behavior. Frontiers in Psychology.

[B55-behavsci-15-00028] Tha S. (2017). Coping with fear of failure: Experiences of young start-up entrepreneurs. Ph.D. thesis.

[B56-behavsci-15-00028] Tornikoski E., Maalaoui A. (2019). Critical reflections–The Theory of Planned Behaviour: An interview with Icek Ajzen with implications for entrepreneurship research. International Small Business Journal.

[B57-behavsci-15-00028] Vaillant Y., Lafuente E. (2007). Do different institutional frameworks condition the influence of local fear of failure and entrepreneurial examples over entrepreneurial activity?. Entrepreneurship and Regional Development.

[B58-behavsci-15-00028] Vamvaka V., Stoforos C., Palaskas T., Botsaris C. (2020). Attitude toward entrepreneurship, perceived behavioral control, and entrepreneurial intention: Dimensionality, structural relationships, and gender differences. Journal of Innovation and Entrepreneurship.

[B59-behavsci-15-00028] Wales W. J., Gupta V. K., Mousa F. T. (2013). Empirical research on entrepreneurial orientation: An assessment and suggestions for future research. International Small Business Journal.

[B60-behavsci-15-00028] Wang J. S. H., Abe A., Kang J. Y., Ku I., Ng I. Y., Peng C., Zhao X. (2024). Social safety net features in East Asia: A comparative analysis using the model family approach. International Journal of Social Welfare.

[B61-behavsci-15-00028] Welpe I. M., Spörrle M., Grichnik D., Michl T., Audretsch D. B. (2012). Emotions and opportunities: The interplay of opportunity evaluation, fear, joy, and anger as antecedent of entrepreneurial exploitation. Entrepreneurship Theory and Practice.

